# The Potential Role of an Artificial Intelligence-Driven Tool in Decision-Making for Mitral Valve Repair Surgery

**DOI:** 10.3390/jcm15062300

**Published:** 2026-03-17

**Authors:** Serdar Akansel, Martina Dini, Simon H. Sündermann, Emilija Myskinite, Stephan Jacobs, Volkmar Falk, Jörg Kempfert, Markus Kofler

**Affiliations:** 1 Department of Cardiothoracic and Vascular Surgery, Deutsches Herzzentrum der Charite (DHZC), 13353 Berlin, Germany; martina.dini@dhzc-charite.de (M.D.); simon.suendermann@dhzc-charite.de (S.H.S.); emilija.miskinyte@dhzc-charite.de (E.M.); stephan.jacobs@dhzc-charite.de (S.J.); volkmar.falk@dhzc-charite.de (V.F.); joerg.kempfert@dhzc-charite.de (J.K.); markus.kofler@dhzc-charite.de (M.K.); 2 Department of Cardiovascular Surgery, Charité-Universitätsmedizin Berlin, 10117 Berlin, Germany; 3 DZHK (German Center of Cardiovascular Research), Partner Site, 10117 Berlin, Germany; 4 Department of Health Sciences and Technology, ETH Zurich, 8057 Zürich, Switzerland

**Keywords:** mitral valve repair, ring sizing, computed tomography, artificial intelligence, ring size prediction

## Abstract

**Background:** Annuloplasty ring sizing is critical for durable outcomes in surgical mitral valve repair (MVr). However, there is no clear consensus on optimal sizing strategies. Artificial intelligence (AI)-based imaging tools may help to reduce uncertainty in preoperative decision-making by providing objective, reproducible and reliable measurements. This study evaluated the predictive capability of a fully automated, computed tomography (CT)-based AI-driven tool for annuloplasty ring sizing in patients undergoing minimally invasive MVr (MI-MVr). **Methods:** A total of 71 consecutive patients undergoing MI-MVr for Carpentier type II mitral valve insufficiency during the study period were included. Preoperative CT scans were analyzed using a cloud-based, fully automated AI tool to quantify mitral valve geometric parameters. Correlations between AI-derived measurements and implanted ring sizes were assessed using the Pearson correlation test. Univariable and multivariable linear regression analyses were performed to identify independent predictors of ring size selection. **Results:** Several AI-derived parameters correlated significantly with implanted ring size, with the strongest correlations observed for commissural width (R = 0.693, *p* < 0.001) and mitral annular area (R = 0.693, *p* < 0.001). In multivariable regression analysis, these parameters were the strongest predictors of annuloplasty ring size (R^2^ = 0.504, *p* < 0.001). Using this model, accurate annuloplasty ring sizing could be predicted in 78.8% of patients. There were no in-hospital mortality and residual mitral regurgitation at discharge. **Conclusions:** A fully automated, CT-based AI-driven tool demonstrated good accuracy for preoperative annuloplasty ring size prediction in MI-MVr and may have the potential to support surgical decision-making, reduce operator dependence, and improve reproducibility.

## 1. Introduction

Artificial intelligence (AI) is increasingly integrated into daily medical practice, promising improvements in efficiency, risk stratification, surgical planning, and postoperative care [1]. Its role may be particularly prominent in preoperative decision-making by advancing the analysis of patient- and intervention-specific factors, thereby improving surgical precision [2]. AI may be especially beneficial in improving several procedural steps of surgical mitral valve repair (MVr), which, at the present moment, is still dependent on surgeons’ experience and case-specific judgment rather than objective and standardized criteria [3,4].

Appropriate annuloplasty ring size selection is critical for durable outcomes in surgical MVr [5]. However, there is no universally accepted consensus on how the ring sizing should be performed. Some experts advocate relying on the anterior mitral leaflet (AML) surface area, while others recommend using the intertrigonal distance and the AML height as sizing parameters [4,6,7]. The potential of AI-based imaging tools to support clinicians during surgical MVr is still underexplored.

This study investigates the capability of a fully automated, computed tomography (CT)-based AI tool (Heart.ai, LARALAB GmbH, Munich, Germany) regarding the adequate ring size prediction in patients undergoing MI-MVr.

## 2. Materials and Methods

### 2.1. Patient Population

**Figure 1:**
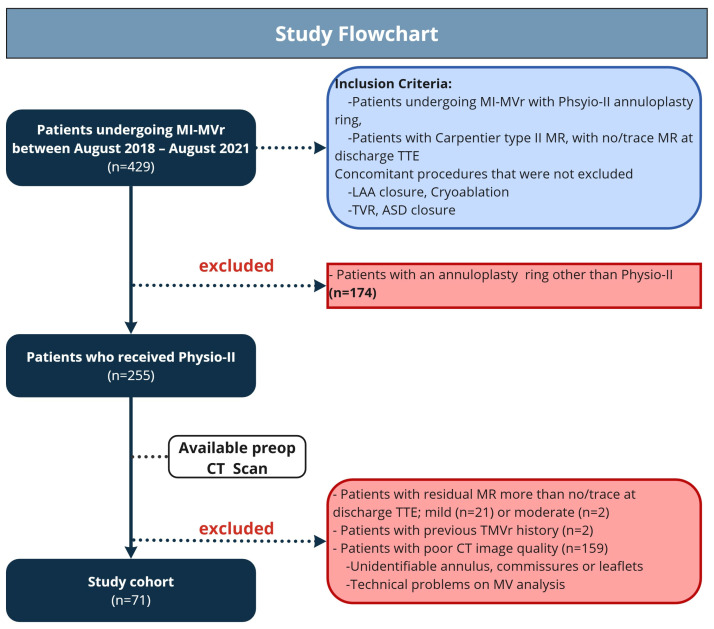
Study flowchart showing study cohort with inclusion and exclusion criteria. MI-MVr = minimally invasive mitral valve repair; MR = mitral regurgitation; TTE = transthoracic echocardiography; LAA = left atrial appendage; TVR = tricuspid valve repair; ASD = atrial septal defect closure; TMVr = transcatheter mitral valve repair; CT = computed tomography.

During the study period between August 2018 and August 2021, 71 patients undergoing minimally invasive MVr (MI-MVr) due to severe MV regurgitation based on a Carpentier type II mitral valve (MV) pathology were included. As shown in the study flowchart, patients who underwent ring annuloplasty using a ring other than the Physio-II (Edwards Lifesciences, Irvine, CA, USA) and those with no/trace residual mitral regurgitation (MR) on discharge transthoracic echocardiography (TTE) were excluded (Figure 1). Additionally, patients with a history of prior transcatheter MVr, which may complicate surgical repair and valve evaluation using the presented tool, were also excluded [8]. Patients with poor CT image quality that did not meet the tool’s technical requirements were not considered for the analysis. The remaining patients were scheduled for MV surgery in accordance with current guidelines [9].

### 2.2. Surgical Procedure

MI-MVr was performed in the study cohort as our clinical standard approach for MV pathologies. The minimally invasive approach was either video-assisted or fully endoscopic using a three-dimensional (3D) endoscope and through a 4 cm right anterolateral minithoracotomy in the fourth intercostal space, as summarized in previous reports [10,11]. Cardiopulmonary bypass (CPB) was established in all individuals by cannulation of the femoral artery and vein using an ultrasound-guided percutaneous or cut-down technique, except for those at risk of retrograde embolization. A preoperative CT scan was routinely performed to define thoracic and vascular access, using 3D reconstruction of the vascular anatomy. Cardiac arrest was achieved using an antegrade cold Bretschneider`s cardioplegia following aortic cross-clamp using a transthoracic Chitwood or an endoaortic balloon occlusion device (IntraClude System, Edwards Lifesciences, Irvine, CA, USA).

Our institutional ring-sizing strategy was based on the AML length and commissural width (CW), measured with manufacturers’ sizers. In cases of discrepancy between measurements, the AML length was considered as the primary determinant for ring size selection.

### 2.3. MV Analysis Using the Heart.ai Tool

**Figure 2:**
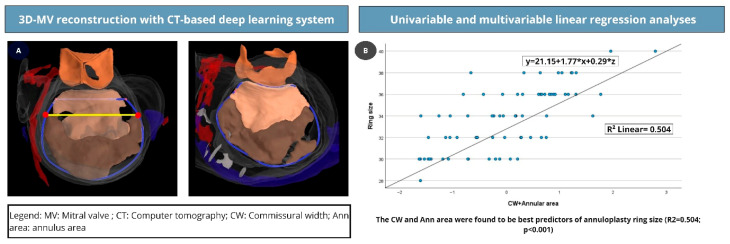
3D reconstruction of the MV using the Heart.ai tool (**A**); multivariable regression model for prediction of implanted ring size using CW and annular area (**B**).

Preoperative CT scans were analyzed using Heart.ai (LARALAB GmbH, Munich, Germany) (https://research.eu.heart.ai/overview, accessed on: 20 January 2026), a cloud-based platform that performs fully automated cardiac CT analysis based on deep learning. The scans were uploaded to Heart.ai and were subsequently automatically processed and analyzed by convolutional neural networks. These networks have been trained on annotated multi-slice CT data from various geographical sources, patient populations, CT scanners and qualities, pathologies and indications. The results are standardized and reproducible segmentations of cardiac chambers and valves, and derived quantitative anatomical and volumetric parameters throughout the cardiac cycle (Figure 2A). All results were available for visual inspection in an integrated web-based DICOM viewer with dedicated multi-planar and three-dimensional reconstructions and views. Quantitative values were exported via a built-in exporting function. For mitral valve assessment, dedicated algorithms segmented the left-sided chambers and mitral leaflets, reconstructed the annular geometry, and computed valve-specific dimensions. The automatically extracted parameters included annular area, commissural width (CW), intertrigonal distance (ITD), and anteroposterior (AP) diameter. In addition, the anterior mitral leaflet (AML) length and AML surface area were quantified (Supplementary Figure S1).

### 2.4. Statistical Analysis

All analyses were made using IBM SPSS Statistics 27.0 for Windows (IBM Corp, Armonk, NY, USA). Continuous variables were presented as mean and standard deviation or median and interquartile range (IQR; 25th–75th percentile). Categorical variables were listed as absolute numbers and corresponding percentages. The Pearson correlation method was used to test the correlation between the Heart.ai tool variables and implanted ring sizes. Univariable and multivariable linear regression were performed for predictive analyses. Variables with a *p*-value < 0.2 in univariable linear regression and those with the highest correlation coefficients in the correlation matrix were considered for the multivariable model. A *p*-value of <0.05 was considered statistically significant.

## 3. Results

### 3.1. Baseline Characteristics

The baseline characteristics of the patients are summarized in Table 1. A total of 71 consecutive patients (47 men, 66.1%) with a mean age of 60.9 ± 14.4 years were scheduled for MI-MVr surgery. The mean EuroScore II and Society of Thoracic Surgeons (STS) scores were 0.99 ± 0.64% and 0.74 ± 0.78%, respectively. The patients had a preserved left ventricular ejection fraction (LVEF), with a mean value of 58.3% ± 5.7.

### 3.2. MV Pathology and Main Echocardiographic Findings

The predominant pathology was posterior mitral leaflet (PML) prolapse or flail (*n* = 56, 78.8%), whereas AML prolapse or flail was present in 11 patients (*n* = 15.4%). Four patients had bileaflet pathology, characterized by a combination of leaflet prolapse and/or flail (Table 2).

The mean C-Sept (coaptation-to-septum) distance was 3.0 ± 0.5 cm and was above 2.5 cm in 58 patients (81.7%).

### 3.3. MVr Techniques 

Our institutional MVr approach is based on the “respect rather than resect” principle. Accordingly, the primary repair techniques included neochordae implantation and cleft closure in case of prominent indentation or cleft, as summarized in Table 3. The most frequently implanted ring size was 36 (*n* = 17, 23.9%) (Supplementary Figure S2).

The CPB was installed via cannulation of the femoral artery and vein using either the percutaneous (*n* = 27, 38.0%) or cut-down technique (*n* = 42, 59.1%), whereas the right axillary artery was used as the vascular access in two patients (2.8%) at risk of retrograde embolization.

### 3.4. Correlation Between the Implanted Ring Size and Heart.ai Tool Findings

**Figure 3:**
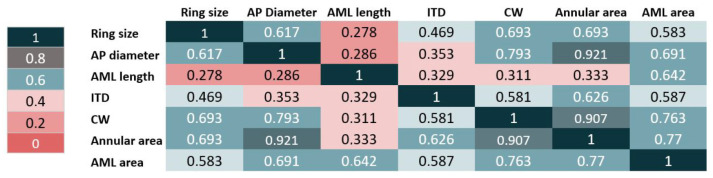
Correlation matrix between the Heart.ai tool-derived parameters and implanted ring size.

The Pearson correlation test evaluated the correlation between the parameters obtained from the tool and the implanted ring size (Figure 3 and Supplementary Table S1). The highest correlation coefficients were provided by CW (R = 0.693; *p* < 0.001), mitral annular area (R = 0.693; *p* < 0.001), AP diameter (R = 0.617; *p* < 0.001), AML area (R = 0.583; *p* < 0.001), and ITD (R = 0.469; *p* < 0.001).

### 3.5. The Potential Role of the Heart.ai Tool in Decision-Making

The predictive ability of the correlated variables, including CW, annular area, AML area, AML length, ITD, and AP diameter, was evaluated using linear regression analysis. Variables with a *p*-value < 0.2 in univariable analyses were subsequently included in the multivariable regression analysis. The variables with the highest correlation coefficients, namely CW (0.693; *p* < 0.001) and annular area (0.693; *p* < 0.001), were used to build the final multivariable regression model (y = 21.135 + 1.777 × X1 +0.290 × X2; R^2^ = 0.504, *p* < 0.001) (Table 4 and Figure 2B).

The greatest level of agreement was observed with the CW and annular area. Additionally, 78.8% of patients received a ring within one size of the predicted ring sizes.

### 3.6. Postoperative Course and Early Outcomes

All patients were discharged without residual MR and SAM, with a mean transmitral gradient of 2.72 ± 1.16 mmHg. There was no in-hospital death (Table 5). The freedom from re-MVr was 98.5% at the one-year follow-up. One patient required re-operation due to subacute MV endocarditis.

## 4. Discussion

The present study aimed to investigate the potential role of a fully automated, CT-based AI tool in pre-MVr decision-making by evaluating its predictive capability for appropriate ring size selection in a MI-MVr cohort.

The main findings from the present study are: (1) The parameters obtained from the AI-driven tool were well correlated with the implanted ring size. (2) In multivariable regression analysis, the CW and annular area showed the highest correlation with annuloplasty ring size. (3) The highest level of agreement was observed with the multivariable regression model, including the CW and annular area. (4) Using the developed model, the implanted ring size could be appropriately predicted within one ring size in 78.8% of patients.

The role of AI in medical practice has been evaluated in numerous studies, with promising results demonstrating improvements in preoperative risk assessment and planning, surgical precision, and outpatient telemonitoring [2,12,13]. Given the essential role of preoperative screening in patients undergoing MI-MVr, AI-driven tools may play a crucial role in enhancing preoperative assessment and supporting decision-making.

Preoperative CT scanning is used to define vascular and surgical access, thereby reducing the risk of retrograde aortic embolization and postoperative stroke [14]. Moreover, preoperative echocardiography provides insight into the underlying pathology and supports the selection of appropriate repair techniques. The lack of consensus on annuloplasty ring sizing strategy, which is critical for durable outcomes in MVr, has increased interest in image-based decision support tools. Several image-based echocardiographic algorithms have been recently reported to assist surgeons in decision-making regarding ring sizing and repair complexity [10,15]. Chikwe et al. demonstrated that 3D-TEE parameters can predict repair complexity [15], while Akansel et al. developed an echocardiographic image-based algorithm incorporating CW and ITD that can predict annuloplasty ring size [10]. A computer-aided design model derived from 3D-TEE has been reported to support ring sizing, demonstrating good correlation with surgeons’ selection [16,17].

However, these tools are limited by several factors. First, echocardiographic image quality is highly dependent on operator expertise and acquisition technique. Second, the raw imaging data often require offline post-processing using a semi-automated software tool to analyze MV geometry. This process is both operator-dependent and time-consuming, limiting the adoption of the tools in daily surgical practice [18].

In light of the preceding arguments, a fully automated AI-driven tool may substantially improve preoperative screening by accelerating analysis, reducing operator dependence and making it more reproducible. Several AI applications have been developed for the automated identification of multiple cardiac pathologies from echocardiography imaging, as well as the identification of heart failure with preserved ejection fraction using the standard four-chamber view [19]. AI technologies are increasingly providing clear advantages and procedural efficiency in cardiac electrophysiology [20]. In the present study, we have observed that accurate annuloplasty ring sizing could be predicted in 78.8% of patients using a fully automated, CT-based AI-driven tool. This tool may be used as a decision-support aid in surgical MVr, potentially increasing the repair rate. Given the presence of outliers, annuloplasty ring sizing should not be performed solely by an automated tool but rather used to support surgical decision-making. Early postoperative outcomes demonstrate that the surgical repairs were technically successful, rather than implying the validation of the AI model. Several additional factors may affect the selection of an accurate ring size, including the risk of systolic anterior motion, the PML length and the length of implanted neochordae loops [10,15,16].

The discrepancy between the investigated tool and previously reported TEE image-based algorithms for ring sizing that rely on ITD and AML length may be related to technical challenges in accurately defining the aorto-mitral curtain, potentially leading to measurement errors [10,21].

However, there are several ethical and data privacy protection concerns related to AI-driven tools [22,23]. The increasing integration of AI-supported decision-making into clinical practice underscores the need for a moral and legal framework that clearly defines responsibility for patient outcomes, as well as data privacy and security [24].

This study has the following limitations. First, the retrospective, single-center design is associated with the disadvantages of such analyses. Second, the findings are restricted to a single annuloplasty ring model and a specific MV pathology. Third, the exclusion of a substantial number of patients due to the absence or low quality of a preoperative CT scan may limit the extrapolation of our findings to the broader patient population. The exclusion of patients with more than trace residual mitral regurgitation on discharge echocardiography may introduce selection bias. Additionally, the inclusion of only patients undergoing a minimally invasive surgical approach may further affect the generalizability of the results. The validation of the developed model against an operator-dependent reference standard represents an inherent limitation, even though the annuloplasty sizing strategy is institutionally standardized. Technical challenges in accurately defining the aorto-mitral curtain may potentially lead to measurement errors of AML length. Further randomized, multicenter prospective studies with larger cohorts, including diverse MV pathologies and annuloplasty ring models, are needed to validate these findings and examine the utility of AI-driven tools in surgical decision-making. Moreover, a structured analysis linking prediction error magnitude to SAM-related anatomy and postoperative hemodynamics would strengthen safety validation.

## 5. Conclusions

AI-driven tools may contribute to reduce uncertainty in selected procedural steps in surgical MVr, which still depends heavily on surgeon experience and subjective judgment so far. This study demonstrates that a fully automated, CT-based, AI-driven tool can support preoperative annuloplasty ring size selection in patients undergoing MI-MVr. Further research focusing on validation is required before clinical adoption.

## Figures and Tables

**Table 1:** Baseline demographics.

	*n* = 71
Age (years)	60.85 (14.37)
Female, *n* (%)	24 (33.8)
EuroSCORE II (%)	0.99 (0.64)
STS PROM score (%)	0.74 (0.78)
BSA (m^2^)	1.95 (0.21)
LVEF (%)	58.38 (5.77)
NYHA class, *n* (%)	
I	20 (29)
II	35 (50.7)
III	14 (20.3)
IV	0 (0)
HT, *n* (%)	32 (45.1)
DM, *n* (%)	1 (1.4)
Hyperlipidaemia, *n* (%)	23 (32.4)
COPD, *n* (%)	4 (5.6)
CAD, *n* (%)	16 (22.5)
CRF, *n* (%)	12 (16.9)
Previous cardiac surgery, *n* (%)	1 (1.4)
Atrial Fibrillation, *n* (%)	
Paroxysmal	13 (18.3)
Persistent	6 (8.5)
Permanent	1 (1.4)

Data are presented as mean ± SD for continuous variables or *n* (%) for nominal and ordinal variables. EuroSCORE II: European System for Cardiac Operative Risk Evaluation II; STS PROM: Society of Thoracic Surgery predicted risk of mortality, BSA: Body surface area; LVEF: left ventricular ejection fraction; NYHA: New York Heart Association; HT: hypertension; DM: diabetes mellitus; COPD: chronic obstructive pulmonary disease; CAD: coronary artery disease; CRF: chronic renal failure; SD: standard deviation.

**Table 2:** Mitral valve pathology and main echocardiographic findings.

	*n* = 71
LVEDD (mm)	54.81 (7.22)
AML leaflet pathology, *n* (%)	
Prolapse	14 (19.7)
Flail	1 (1.4)
Billowing	2 (2.8)
PML leaflet pathology, *n* (%)	
Prolapse	42 (59.2)
Flail	18 (25.4)
Billowing	1 (1.4)
C-Sept distance (cm)	3 (0.54)
C-Sept > 25 mm, *n* (%)	58 (81.7)

Data are presented as mean ± SD for continuous variables or *n* (%) for nominal and ordinal variables. AML: anterior mitral leaflet; LVEDD: left ventricular end-diastolic diameter; PML: posterior mitral leaflet; SD: standard deviation; C-Sept: coaptation-to-septum distance.

**Table 3:** MVr techniques and operative details.

	*n* = 71
Ring size, median (IQR)	34 (32–36)
Additional MVr procedures, *n* (%)	
AML neochords	11 (15.4)
PML neochords	56 (78.8)
Bileaflet repair	4 (5.6)
Cleft closure	14 (19.7)
Implanted neochordae length (mm)	
AML	20.91 (3.94)
PML	16.64 (4.57)
Concomitant TVr, *n* (%)	4 (5.6)
Percutaneous cannulation, *n* (%)	27 (38)
Arterial cannulation, *n* (%)	
Right femoral artery	57 (80.3)
Left femoral artery	12 (16.9)
Right axillary artery	2 (2.8)
**Operative variables**	
CBP time (min)	119.83 (38.71)
Aortic cross clamp time (min)	74.17 (21.52)

Data are presented as mean ± SD for continuous variables or *n* (%) for nominal and ordinal variables. IQR: Interquartile range; MVr: mitral valve repair; AML: anterior mitral leaflet; PML: posterior mitral leaflet; TVr: tricuspid valve repair; SD: standard deviation.

**Table 4:** Multivariable linear regression analysis using Heart.ai-derived parameters showing a significant association with implanted ring size.

	Univariable Linear Regression Analysis			Multivariable Linear Regression Analysis			
	R^2^	Correlation Coefficient *	*p*-Value	Regression Coefficient	R^2^	Beta	*p*-Value
Constants				21.135	0.504		<0.001
ITD	0.220	0.469	<0.001				
CW	0.480	0.693	<0.001	1.777		0.363	
Annular area	0.480	0.693	<0.001	0.290		0.364	
AML area	0.340	0.583	<0.001				
AP diameter	0.381	0.617	<0.001				
AML length	0.077	0.515	0.019				

* Pearson correlation; ITD: intertrigonal distance; CW: commissural width; AML: anterior mitral leaflet; AP: anterior-posterior.

**Table 5:** Post-repair echocardiographic findings and early outcomes.

	*n* = 71
Mean transmitral gradient (mmHg)	2.72 (1.16)
LVEF grade (%)	
<30%	0 (0)
30–50%	23 (32.4)
>50%	48 (67.6)
In-hospital mortality, *n* (%)	0 (0)
ICU length of stay (h)	42.77 (41.90)
Hospital length of stay (days)	8.70 (6.40)
Revision for bleeding	8 (11.3)
Redo MV repair	0 (0)

Data are presented as mean ± SD for continuous variables or *n* (%) for nominal and ordinal variables. LVEF: left ventricular ejection fraction; ICU: intensive care unit; MV: mitral valve; SD: standard deviation.

## Data Availability

The data obtained from our institutional database cannot be shared publicly due to data protection policies but will be shared with interested parties upon reasonable request to the corresponding author.

## Supplementary Materials

The following supporting information can be downloaded at: https://www.mdpi.com/article/10.3390/jcm15062300/s1, Figure S1: MV analysis using the Heart.ai tool; Figure S2: Number of the implanted rings by size; Table S1: Heart.ai-derived parameters regarding MV geometry.

## Abbreviations

The following abbreviations are used in this manuscript:

AI	Artificial Intelligence
MVr	Mitral valve repair
AML	Anterior mitral leaflet
CT	Computed tomography
MI-MVr	Minimally invasive mitral valve repair
MR	Mitral regurgitation
MV	Mitral valve
TTE	Transthoracic echocardiography
CPB	Cardiopulmonary bypass
CW	Commissural width
ITD	Intertrigonal distance
AP	Anterior-posterior
3D-TEE	Three-dimensional transesophageal echocardiography
LVEF	Left ventricular ejection fraction
PML	Posterior mitral leaflet
C-Sept	Coaptation to septum
