# Supplementary material for: The Potential Role of an Artificial Intelligence-Driven Tool in Decision-Making for Mitral Valve Repair Surgery
Source: J Clin Med. 2026 Mar 17;15(6):2300. doi: 10.3390/jcm15062300 (PMC13026876; doi:10.3390/jcm15062300)

**Figure S1:** MV analysis using the Heart.ai tool

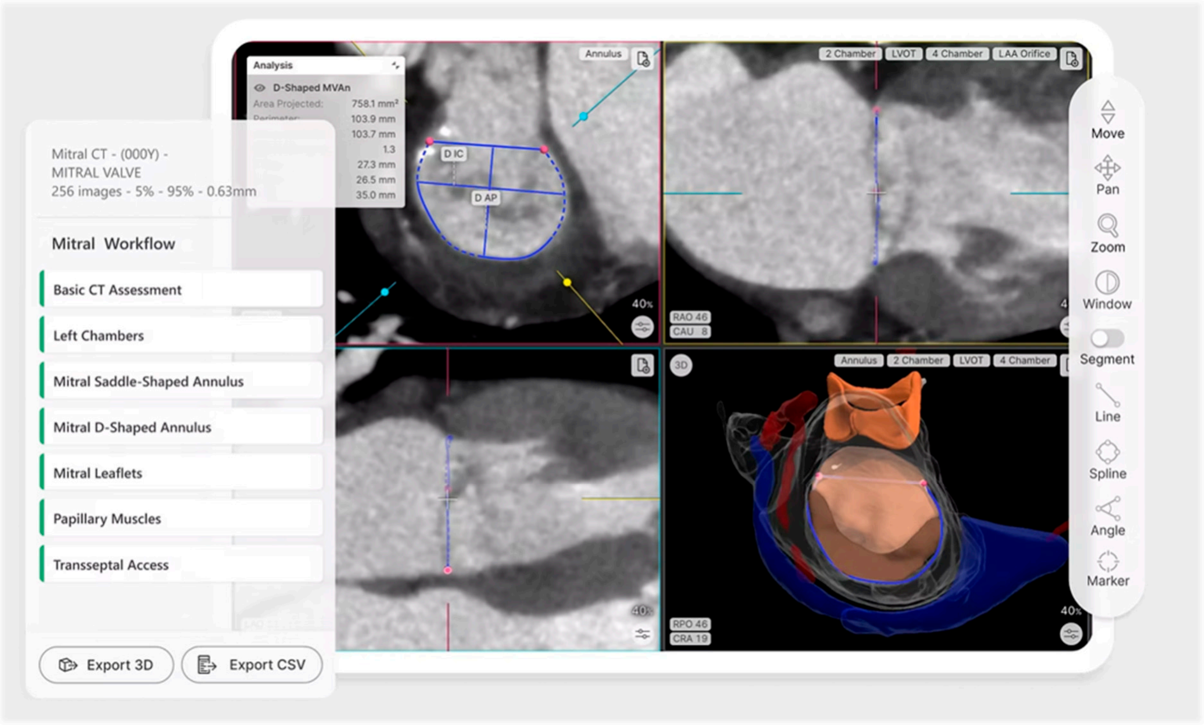


**Table S1:** Heart.ai-derived parameters regarding MV geometry

| | ***n* = 71** |
|---|---|
| C-Sept distance (cm) | 3 (0.54) |
| C-Sept sufficient, *n* (%) | 58 (81.7) |
| AP diameter (cm) | 3.52 (0.54) |
| AML length (cm) | 2.04 (0.43) |
| ITD (cm) | 3.35 (0.45) |
| CW (cm) | 4.60 (0.60) |
| Annulus Area (cm²) | 15.41 (3.66) |
| AML Area (cm²) | 8.38 (2.44) |
| PML length (cm) | 2.21 (0.50) |

Data are presented as mean ± SD for continuous variables or *n* (%) for nominal and ordinal variables.

C-sept: Commissure-to-septum; AP: Antero-posterior; AML: anterior mitral leaflet; ITD: Intertrigonal distance; CW: Commissural width; PML: posterior mitral leaflet; SD: standard deviation.

**Figure S2:** Number of the implanted rings by size

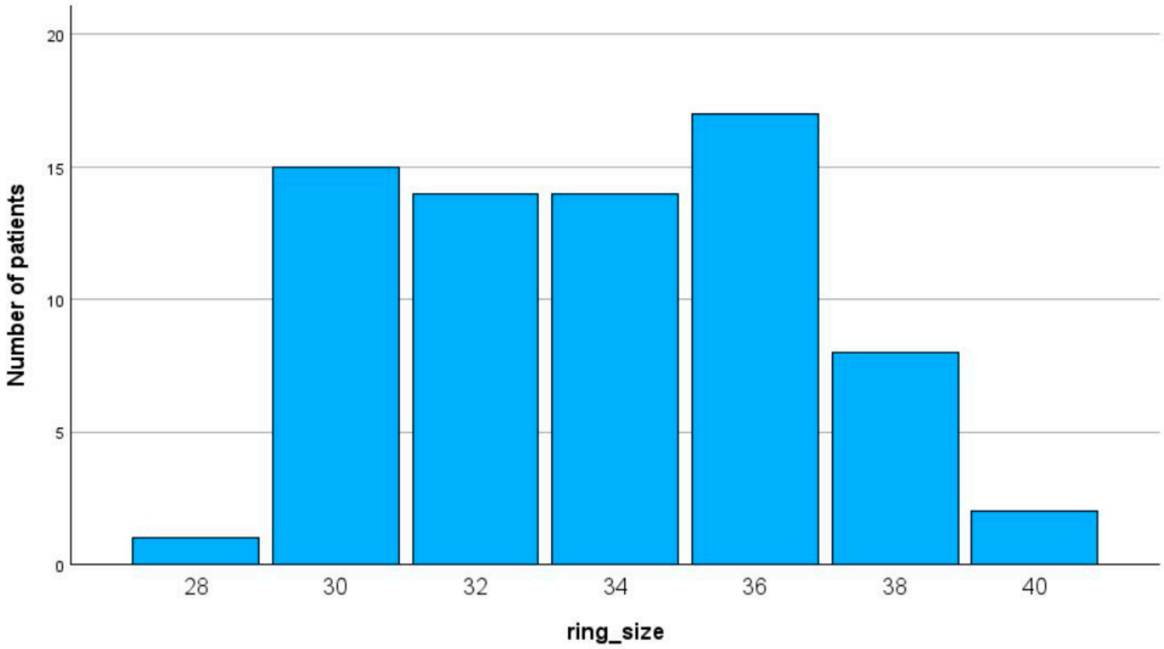

Supplement: Supplementary file 1 [file jcm-15-02300-s001.zip › jcm-4156165-supplementary.pdf]
